# Profiling of fecal analytes as a potential biomarker in rheumatoid arthritis

**DOI:** 10.3389/fimmu.2025.1577590

**Published:** 2025-05-19

**Authors:** Zhiyi Wang, Yujia Shi, Yachen Yang, Bangdong Gong, Jianmin Xie

**Affiliations:** ^1^ Department of Rheumatology, The Second Affiliated Hospital of Nanjing Medical University, Nanjing, China; ^2^ Department of Rheumatology, Tongji Hospital of Tongji University, Shanghai, China

**Keywords:** rheumatoid arthritis, cytokines, biomarkers, intestinal inflammation, intestinal barrier function

## Abstract

**Background:**

Loss of gut barrier integrity has been observed in rheumatoid arthritis (RA). While systemic inflammation in RA has been extensively investigated, intestinal-specific inflammatory processes remain poorly understood. This study is designed to identify a novel biomarker panel combining fecal cytokine profiles with gut barrier biomarkers to discriminate RA patients with varying disease progression.

**Methods:**

Feces (Fc) and plasma (Pl) were obtained from 62 Naive RA patients (NA), 47 remission RA patients (RE), 28 difficult-to-treat RA patients(D2T), and 70 healthy controls (HC). A panel of 12 cytokines and gut barrier markers, including intestinal Fatty-Acid-Binding Protein-2 (FABP2), zonulin, Hypoxia-Inducible Factor-2α (HIF-2α), D-lactate, LBP and fecal calprotectin (FCAL), was quantified by ELISA. Statistical integration with clinical parameters was performed using univariate and multivariate approaches.

**Results:**

NA and D2T patients demonstrated marked elevations in fecal pro-inflammatory cytokines compared to RE and HC groups, including IL-6, Granulocyte-Macrophage Colony-Stimulating Factor (GM-CSF), IL-1 beta (IL-1β), Interferon-gamma (INF-γ), IL-23, Tumor Necrosis Factor-Alpha (TNF-α), IL-21, IL-17A/F, and IL-22. Fecal zonulin and plasma HIF-2α were significantly elevated in both NA and D2T groups, whereas fecal D-lactate showed a pronounced decrease in the NA and D2T groups. These biomarkers demonstrated the strongest correlation with disease severity indices. Receiver operating characteristic (ROC) analysis revealed that fecal FABP2, zonulin and D-lactate exhibited superior discriminative capacity between the NA and RE groups. whereas fecal zonulin showed remarkable diagnostic potential for distinguishing NA from D2T groups compared to plasma counterparts. The discriminant scores (DS) model incorporating fecal zonulin and plasma HIF-2α demonstrated superior discriminatory performance between the D2T and NA groups compared to the model utilizing the top five plasma parameters.

**Conclusions:**

Our fecal profiling methodology provides novel insights into the gut mucosal cytokine microenvironment during RA progression. The dissociation between fecal and plasma inflammatory profiles underscores the critical importance of localized gut immune monitoring in RA management.

## Introduction

RA is classified as an autoimmune disorder marked by the destruction of joints and additional manifestations beyond the joints (1). The onset of RA results from a complex interaction among various cytokines and immune cells that facilitate the growth of synoviocytes, leading to the degradation of cartilage and bone (2). Recent research has demonstrated that dysbiosis compromises the intestinal barrier, heightens intestinal permeability, exposes immune cells to more bacterial antigens, and causes inflammatory responses in both the intestines and joints (3–5). The disruption in gut microbiota, coupled with the spread of harmful gut bacteria and their byproducts are key factors in the dysfunction of the “gut-joint axis” (6–8). This hypothesis underscores the link between the gut and joints, indicating that impairment of the gut barrier significantly contributes to the pathogenesis of RA (8). Pathogenic bacteria and fungi residing in the gut can closely stick to the intestinal wall, possibly damaging the barrier and affecting the body’s immune response (9). Furthermore, recent findings suggest that intestinal inflammation and heightened permeability frequently precede flare-ups of RA, with affected people showing higher intestinal permeability, movement of bacterial parts into the bloodstream, and a rise in inflammatory markers, all of which link to more severe clinical symptoms (4, 5, 10–12). However, there is limited understanding of the relationship between gut inflammation, barrier dysfunction, and systemic inflammation in RA, particularly regarding their effects on clinical activity during the acute phase. Consequently, further investigation into the connections between the gut barrier and RA is warranted.

Intestinal permeability serves as a crucial diagnostic indicator of the integrity of the intestinal barrier and is routinely employed in the evaluation of mucosal injury across multiple gastrointestinal diseases, including celiac disease and Crohn’s disease (13). When the gut barrier is damaged, proteins like albumin can leak from blood vessels into nearby tissues and eventually into the gut (13). As a result of this process, fecal albumin has emerged as a promising biomarker for assessing intestinal permeability. Moreover, research has shown that various cytokines—including IL-17A, IL-17F, IL-6, IL-23, IL-21, IL-22, IL-4, IFN-γ, IL-10, IL-1β, GM-CSF, and TNF-α—exhibit a close association with the host’s anti-microbial immunity (9, 14). These cytokines play a significant role in mediating immune responses, highlighting their importance in both the maintenance of intestinal barrier function and the overall immune defense against pathogens. In clinical practice, profiling circulating cytokines has been shown to correlate with prognosis across various stages of RA, facilitating the differentiation between patients exhibiting disease activity and those who do not, while also influencing clinical outcomes such as mortality (15). However, there is little consensus regarding which of several endogenous proteins serve as reliable indicators of intestinal inflammation at different stages of RA. Furthermore, our understanding of localized gut inflammation and the critical immunological processes that affect barrier disruption in RA remains limited, particularly concerning the relationships between similar cytokine markers and clinically significant outcomes. Here, we conducted this study to explore biomarkers that characterize RA prognosis through fecal cytokines.

The aim of this study is to develop a method for characterizing and differentiating intestinal mucosal inflammation and damage in RA patients during active disease or D2T phases, utilizing serum and fecal samples as biological substrates. In this context, a group of cytokines and markers of gut permeability were examined in both feces and plasma from RA. We assessed and compared plasma and fecal levels of inflammatory cytokines, zonulin, HIF-2α, FABP2, and D-lactate, as well as Pl-LBP and FCAL, as biomarkers of intestinal barrier integrity and inflammation, whilst their association with prognosis across various stages of RA was investigated.

## Materials and methods

### Participant data and biological sampling

The study, approved by the Ethics Committee of the Second Affiliated Hospital of Nanjing Medical University, complied with ethical guidelines. Written informed consent was obtained from 137 patients or their representatives (if incapacitated) within 48 hours, with personal data protected. From April 2021 to March 2023, patients were categorized into three groups: NA (n=62), RE (n=47), and D2T (n=28). Inclusion criteria for the 62 early RA patients: (1) no prior treatment with csDMARDs, bDMARDs, tsDMARDs, or GCs; and (2) a Disease Activity Score 28 (DAS28) between 2.6 and 5.1. After a patient is admitted to the hospital, we collect patient information, including measurements of CRP, ESR, Rheumatoid Factor, and anti-CCP Autoantibodies (RF and anti-CCP) are measured by ELISA; inflammatory parameters (CRP and ESR) are detected by an automated analyzer.

The 47 patients in the RE group had received antirheumatic drugs and achieved remission (DAS28 < 2.6). In this trial, RA patients in the D2T group were defined by three criteria: ①,Failure to reach the treatment goal of at least low-disease activity after 3–6 months of treatment with two conventional synthetic DMARDs (csDMARDs) combined with one bDMARD or one tsDMARD; ②, DAS 28 using erythrocyte sedimentation rate (DAS28 - ESR) > 5.1 with signs of inflammatory disease activity; ③, Inability to reduce glucocorticoid (GC) dose to 10 mg/day of prednisone or equivalent. The inclusion criteria were: I, Meeting the 2010 American College of Rheumatology (ACR)/European League Against Rheumatism (EULAR) classification guidelines for RA (16); II, Having complete clinical data; III, Being assessed by DAS28. The exclusion criteria are: i. Combination of hepatic, renal or other major organ dysfunction; ii. Combination of gastrointestinal diseases (e.g., inflammatory bowel disease and peptic ulcer, etc.) and gastrointestinal surgeries (e.g., gastrectomy, colectomy, and bariatric surgery); iii. Combination of malignant tumors; iv. Combination of infectious diseases, such as hepatitis B and pneumonia; v. Combination of other rheumatic diseases; vi. The patient is a pregnant or lactating woman; vii. History of mental illness. There were no statistically significant differences in age or gender composition between the four groups. Demographic, clinical, biochemical, and medication data were collected at the time of sampling, with detailed information provided in **Table 1**.

**Table 1:** Summary of clinical characteristics of study groups.

Parameter	Indexes	Naive group	Remission group	D2T group	P value
Number per group		62	47	28	
Age (year)	Median (IQR)	57 (42-62)	52 (40-62)	59 (44-64)	NS
Gender (M:F)		30/32	23/24	13/15	NS
BMI (kg/m^2^)	Median (IQR)	25.23 (19.21-32.63)	26.12 (20.60-33.24)	26.79 (20.04-34.73)	NS
Duration (months)	Median (IQR)	1 (0-6)	5 (1-7)	12 (5-20)	p < 0.01
Biochemistry					
CRP mg/dL	Median (IQR)	0.22 (0.13-0.27)	0.15 (0.06-0.18)	0.35 (0.27-0.4)	p = 0.047
ESR mm/h	Median (IQR)	42.7 (30.8-50.3)	18.77 (12.65-20.11)	75.75 (55-96.29)	p < 0.01
RF U/ml	Median (IQR)	368.5 (334.1-391.7)	246.7 (209.8-257.9)	399.5 (367-427.18)	p < 0.01
Anti-CCP RU/ml	Median (IQR)	96.2 (86.3-104.5)	73.1 (52.8-89.7)	108.9 (91.6-123.8)	p = 0.049
DAS28	Median (IQR)	3.8 (2.6-5.0)	2.3 (1.9-2.5)	5.4 (5.3-5.8)	p < 0.01
Medications					
NSAIDs	n (%)	24 (38.71)	19 (40.43)	13 (46.43)	NS
Ongoing csDMARDs	n (%)	Na	45 (95.74: MTX, n=30; LEF, n=9; HYD, n=4; SUL, n=2)	22 (78.57: MTX, n=11; LEF, n=9; HYD, n=1; SUL, n=1)	p = 0.021
Ongoing bDMARDs	n (%)	Na	20 (42.55: TNFi, n=10; IL-6i, n=9; RTX, n=1	25 (89.28: TNFi, n=10; IL-6i, n=8; RTX, n=7	p < 0.01
Ongoing tsDMARDs	n (%)	Na	5 (10.64 JAKi)	26 (92.86 JAKi)	p < 0.01
Glucocorticoids	n (%)	Na	15 (31.91)	24 (85.71)	p < 0.01
Glucocorticoids, (mg/day)	Median (IQR)	Na	3 (0-8)	14 (11-19)	p < 0.01

D2T group, Difficult-to-treat group; NA, not applicable; NS, not significant; TNFi, TNF Inhibitor (etanercept, adalimumab, infliximab, golimumab, certolizumab pegol); IL-6i:IL-6 Inhibitor (tocilizumab, sarilumab); RTX, rituximab; MTX, methotrexate; LEF, leflunomide; HYD, hydroxychloroquine, SUL, sulfasalazine; JAKi, JAK inhibitors (tofacitinib, baricitinib, upadacitinib). p values: p values were obtained from Kruskal-Wallis test or Chi-square tests.

### Laboratory assessments

Biological samples (plasma and stool) were collected from each study participant using untreated sterile universal test tubes for stool and sodium heparin test tubes for blood. Fecal lysates (FLs) were prepared from frozen stool samples via chemical and mechanical homogenization (9). ELISA kits for IL-1β, IL-6, TNF-α, IL-21, IL-10, IL-4, and INF-γ were purchased from Thermo Scientific, Monza, Italy. Kits for IL-17A/F, IL-22, IL-23, GM-CSF and FABP2 were from R&D Systems, Milano, Italy. These cytokines were selected based on available research evidence. Kits for HIF-2α were from MyBioSource, California, the United States of America. Kits for D-lactate were from Creative Diagnostics, New York, the United States of America. Commercially available ELISA kits from Immundiagnostik AG, Bensheim, Germany, were used to measure zonulin and FCAL. Plasma LBP levels were measured using an LBP ELISA kit (Hycult Biotech, Netherlands). ELISA for specific markers was performed following the manufacturer’s instructions.

### Statistical analysis

Statistical analyses included the Mann-Whitney U test or the Kruskal-Wallis test with Dunn′s correction for group comparisons and Chi-square test for categorical data. Multiple testing was adjusted using the Benjamini-Hochberg false discovery rate (FDR) algorithm. The results of the Kruskal-Wallis test were denoted as “KWp” in the text, graphs, and tables, respectively, while the FDR-adjusted q-values are denoted as “BHq”. Correlations were assessed using Pearson′s coefficients with Bonferroni family-wise error rate (FWER) correction. Multivariate analyses included unsupervised principal component analysis (PCA) and supervised orthogonal projections to latent structures discriminant analysis (OPLS-DA). Cross-validation and permutation test were used to assess the stability of the OPLS-DA model (R2Y: fitness of model, Q2: predictive capability). Variable importance in the projection (VIP) and fold change (FC) were calculated in the OPLS-DA model. P values were estimated with Wilcoxon rank-sum test on Single dimensional statistical analysis. Statistically significant among groups were selected with VIP > 1, FC > 2 or < 0.5, and p values < 0.05. Receiver operating characteristic curves (ROC) were then constructed by plotting sensitivity versus specificity curves. Area under the curve (AUC) values of the ROC curves were calculated to estimate the accuracy of these potential biomarkers in distinguishing NA from RE and D2T from NA.

## Result

### Measurement of fecal and plasma cytokines and gut barrier markers in RA patients and controls

**Figure 1:**
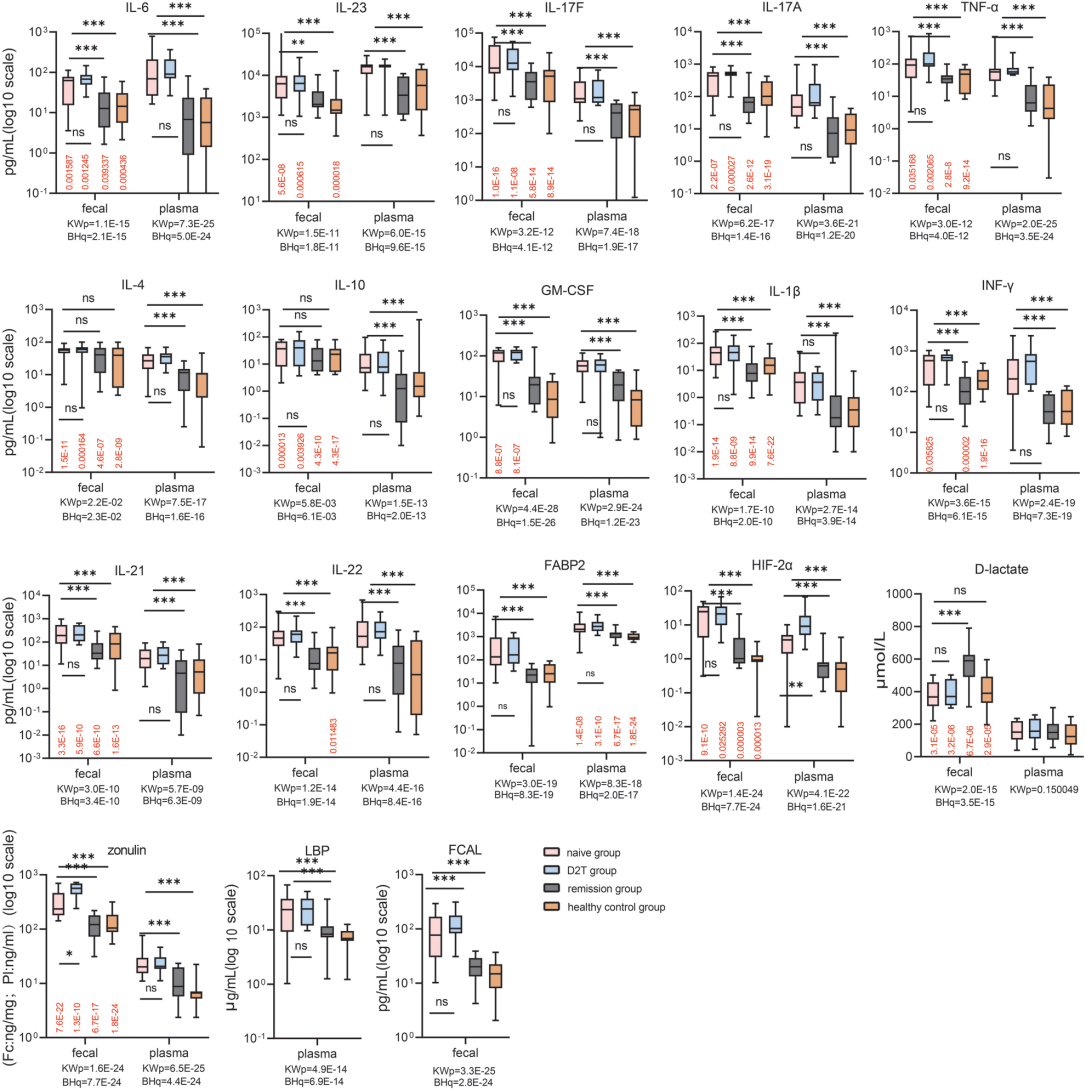
Comparison of fecal and plasma analyte concentrations in the naive, remission, D2T, and healthy control groups. Red values: BH adjusted q values for paired feces vs. plasma (Wilcoxon) comparisons for each group. KWp and BHq values: Kruskal-Wallis p-values and BH-adjusted q-values, Kruskal-Wallis p-values and BH-adjusted q-values used for overall between-group comparisons. Bonferroni-corrected p-values for multiple comparisons when KWp is significant. ns: p ≥ 0.05; *p < 0.05; **p < 0.01; ***p < 0.001. BH, Benjamini-Hochberg; HIF-2α, Hypoxia-Inducible Factor-2 Alpha; FABP2, fatty-acid-binding protein-2; FCAL, fecal calprotectin; LBP, LPS-Binding Protein; IFN-γ, gamma interferon; TNF-α, tumor necrosis factor alpha; GM-CSF, Granulocyte-Macrophage Colony-Stimulating Factor.

*Fecal* and Plasma levels of cytokines and barrier markers were analyzed in RA patients and controls to assess disease severity (**Figure 1**). Fecal FABP2, HIF-2α and FCAL were higher in the NA and D2T groups than in the RE and HC groups (P < 0.001). Similarly, fecal D-lactate levels were lower in the NA and D2T groups than in the RE group (Dunn’s p_NA_ < 0.001; Dunn’s p_D2T_ < 0.001). Interestingly, fecal zonulin was significantly higher in the D2T group than in the NA and RE groups (Dunn’s p = 0.013 and Dunn’s p < 0.001). Similarly, plasma FABP2, zonulin, HIF-2α and LBP levels were higher in NA and D2T groups than in RE and HC groups (p < 0.001). However, plasma D-lactate levels were comparable in all groups (p = 0.15). Notably, only HIF-2α exhibited a significant difference in plasma analytes between the NA and D2T groups (Dunn’s p = 0.007), while the other plasma analytes did not show significant differences between these two groups.

Next, we measured plasma and fecal levels of key pro- and anti-inflammatory cytokines (IL-17A/F, IL-6, IL-21, IL-22, IFN-γ, IL-4, GM-CSF, IL-10, IL-1β, TNF-α, and IL-23) across the four groups. The data showed that the fecal levels of all 10 cytokines (including IL-17A, IL-17F, IL-6, IL-21, IL-22, IFN-γ, IL-1β, TNF-α, GM-CSF and IL-23) measured were higher in the NA and D2T groups than in the RE group and HC (Dunn′s p < 0.05). In contrast, fecal levels of IL-4 and IL-10 did not show significant differences among the four groups (Dunn′s p > 0.05). When analyzing plasma levels of the 12 cytokines, both the NA and D2T groups displayed higher levels compared to the RE and HC groups (Dunn’s p < 0.05). Notably, plasma and fecal levels of these 12 cytokines were comparable between the RE and HC groups, as well as between the NA and D2T groups.

In addition, NSAIDs are known to damage intestinal epithelial cells, and approximately 39% of naive patients use NSAIDs. To explore whether that the use of NSAIDs in the RA groups exerts a significant influence on gut permeability, we assessed gut permeability markers such as zonulin, FABP2, and LBP in plasma and feces of naive RA patients with NSAIDs. The data showed that these permeability markers were higher in the NA group using NSAIDs than in HC. However, when analyzing the NA group of RA patients who excluded NSAIDs, plasma and fecal zonulin, FABP2, and plasma LBP still remained higher than in HC (q < 0.01). The analysis on the RA group of on vs.no NSAIDs hinted that Impaired gut permeability in the NA group might related with disease occurrence (**Supplementary Figure 1**).

### Comparison of paired fecal and plasma analytes in RA patients and controls

To furtherly investigate the discrepancy between the systemic circulation and the intestinal microenvironment, *paired* fecal and plasma analytes from the same patients and controls were compared (**Figure 1**). Among the typical pro- and anti-inflammatory cytokines, cytokines IL-4, IL-10, TNF-α, IL-21, IL-17A/F and IL-1β (and IFN-γ and GM-CSF to a lesser extent), exhibited elevated in feces compared with their plasma counterparts across all groups (p < 0.05). Fecal levels of IL-23 were lower than plasma levels in the NA, D2T and HC groups (p < 0.001). In the NA and D2T groups, fecal IL-6 levels were lower than those in plasma samples (p < 0.05), whereas in the RE and HC groups, fecal IL-6 levels were higher than those in plasma (p < 0.05). Furthermore, in the NA, D2T, and RE groups, the levels of IL-22 were comparable between plasma and fecal samples. Fecal levels of D-lactate, zonulin and HIF-2α in all 4 groups were increased compared with their plasma counterparts, whereas the fecal level of FABP2 was lower than that in plasma (q < 0.05). The marked disparity in analyte levels between fecal and plasma samples suggests that fecal analytes may serve dual pro- and anti-inflammatory functions in gut immunity, and appear to be subject to distinct regulatory mechanisms compared to plasma analytes in rheumatoid arthritis patients.

### Hierarchical cluster analysis of fecal and plasma analytes in RA patients

**Figure 2:**
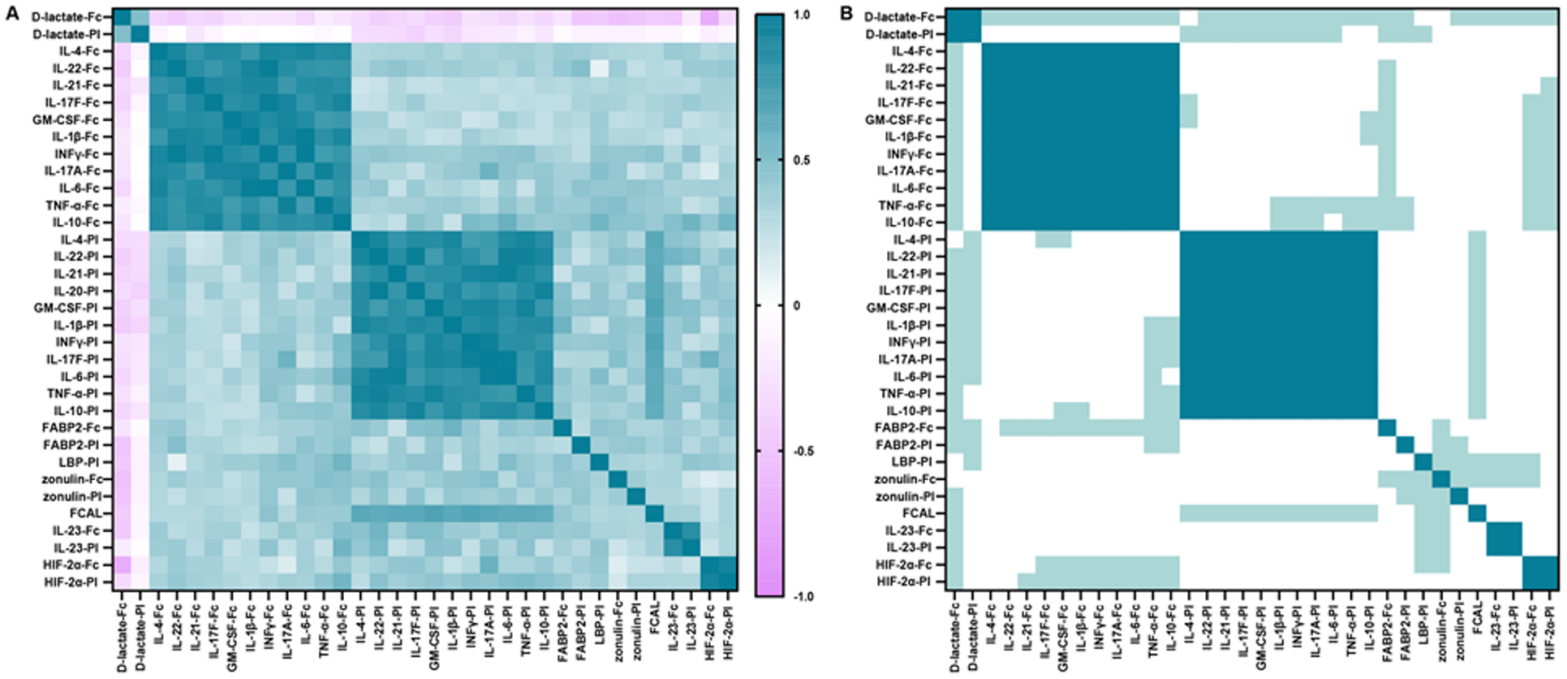
Correlation between fecal and plasma cytokines, FABP2, zonulin, HIF-2α, D-lactate, FCAL and plasma LBP. **(A)** Correlation plot: matrix of interrelationships obtained by hierarchical clustering based on Pearson’s correlation coefficient for assessing correlation between analytes. **(B)** Significance plot: p-values associated with the correlation levels in **(A)**. White, not significant; light green, p < 0.05 (trend); green, p < 0.01 (significant). Significance thresholds were determined by Bonferroni FWER correction.

To explore whether the cytokines production is associated with the shedding of gut integrity markers in inflammatory conditions in RA patients, we performed Pearson’s correlation-based hierarchical clustering on all fecal and plasma analytes from both patients and control groups (**Figure 2**). Three obvious distinct independent co-regulatory clusters were determined. Clusters 1 and 2 included cytokines (IFN-γ, IL-17A, IL-4, IL-22, IL-21, IL-17F, GM-CSF, IL-1β, IL-6, TNF-α, and IL-10) that were categorized based on their source being either fecal or plasma. This result reveals substantial collinearity within each compartment and indicates a scarcity of association among these different cytokines across the two types of matrices. Furthermore, IL-23, HIF-2α, and D-lactate constituted cluster 3, regardless of their anatomical source, emphasizing the influence of these microbial metabolites on the maintenance of intestinal barrier function. Nevertheless, other analytes, including FCAL, LBP, zonulin, and FABP2, did not fit into any clusters, suggesting a modulation mechanism that operates independently of clustering.

### Correlations of fecal and plasma analytes with disease activity in RA patients

**Figure 3:**
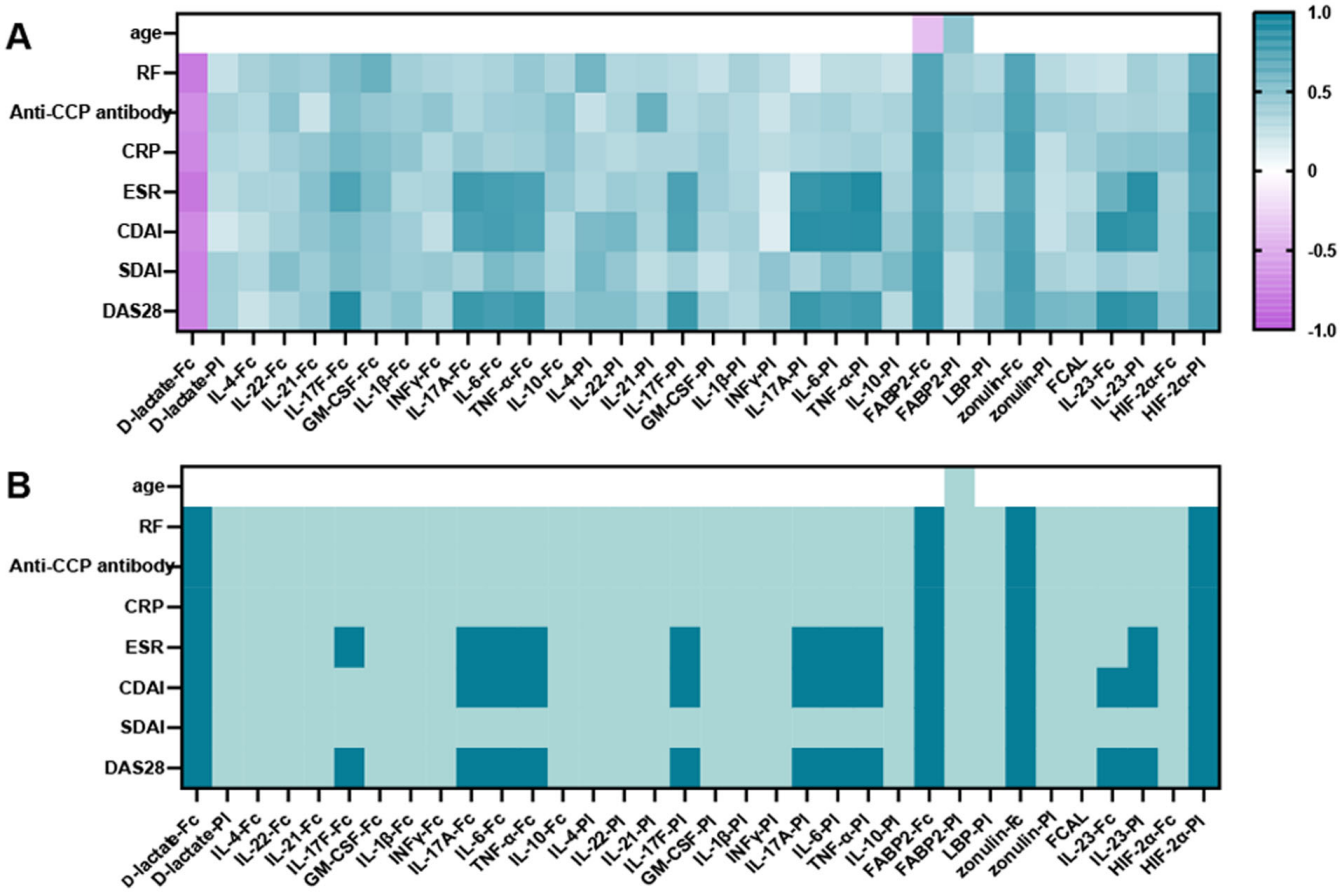
Correlations between analytes (including plasma and fecal cytokines, FABP2, D-lactate, HIF-2α, zonulin, FCAL, plasma LBP) and indicators of disease activity and age. **(A)** Plot of correlation levels of fecal and plasma analytes with each clinical parameter. Using Pearson’s correlation coefficient, only correlation coefficients with p < 0.05 are indicated. **(B)** Significance plot: p-values associated with the correlation levels in **(A)**: white, not significant; light green, p < 0.05 (trend); green, p < 0.01 (significant). Significance thresholds were determined by Bonferroni FWER correction. ESR, Erythrocyte Sedimentation Rate; CRP, C - reactive protein; RF, Rheumatoid Factor; BMI, Body Mass Index; DAS28, Disease Activity Score 28; CDAI, Clinical Disease Activity Index; SDAI, Simple Disease Activity Index.

To determine the relationship between analytes and clinical features, this study demonstrated a trend towards correlations between plasma and fecal analytes with indices of disease activity (including DAS28, SDAI, CDAI, ESR, CRP, RF, and anti-CCP antibody) (**Figure 3**). Plasma and fecal analytes (including LBP, FCAL, IL-21, IL-22, IFN-γ, IL-4, GM-CSF, IL-10 and IL-1β) showed a positive trend of correlation with disease activity indices. except for D-lactate, which showed a negative trend of correlation with these indices. Based on the Bonferroni-corrected critical value of p < 0.05, We observed robust and significant positive correlations between zonulin-Fc, FABP2-Fc and HIF-2α-Pl with these indices of disease activity. Conversely, fecal D-lactate exhibited a statistically significant correlation negatively with these indices (p < 0.05). Notably, plasma and fecal IL-6, TNF-α, IL-23, and IL-17A/F were significantly correlated with DAS28, CDAI and ESR. The data demonstrated a more substantial interdependency between fecal and serum analytes and clinical parameters, positing their prospective applicability as surrogate markers for the evaluation of clinical disease activity.

### Discriminative analysis in RA via profiling of plasma and fecal analytes

**Figure 4:**
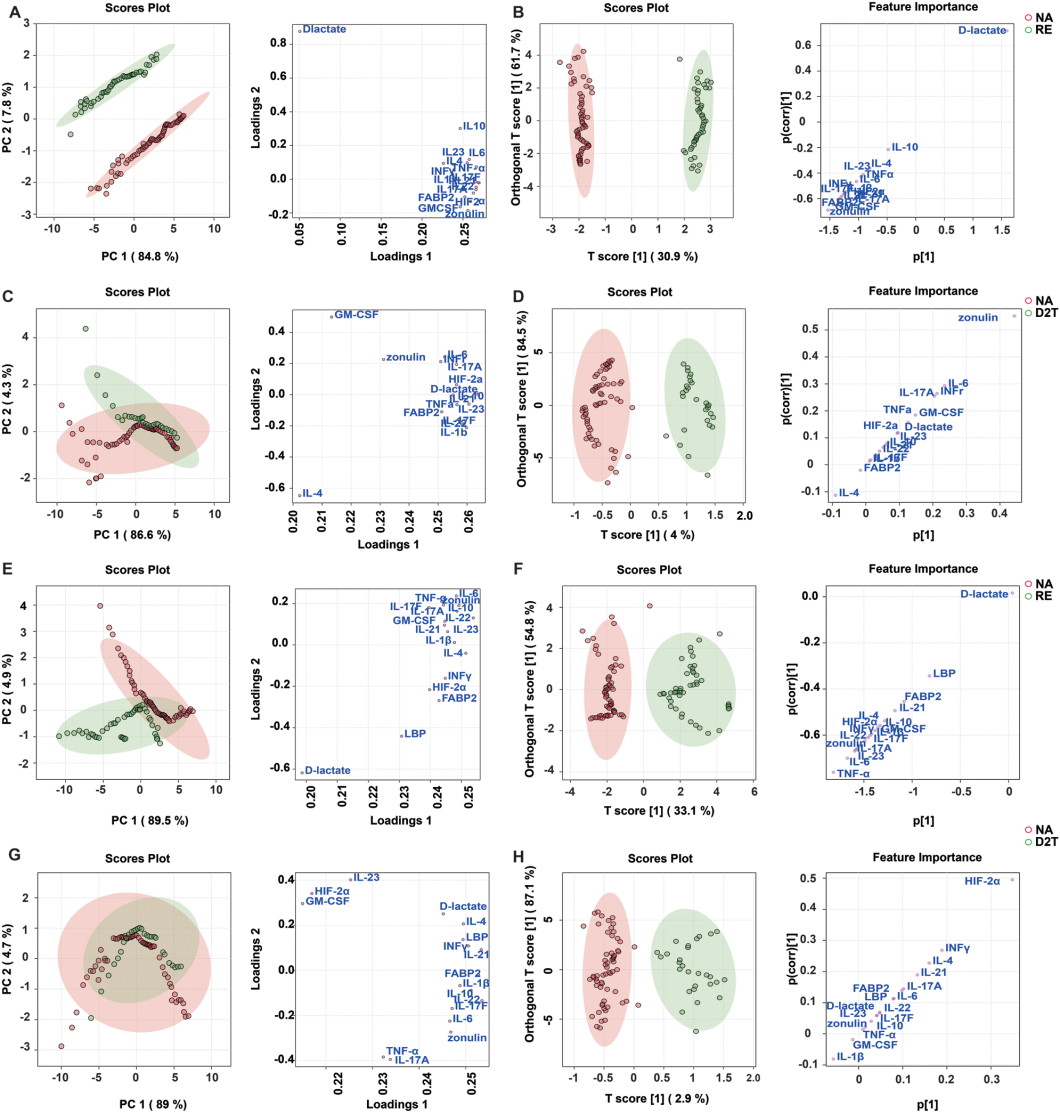
Discrimination between patient groups using fecal and plasma analytes by multivariate analysis by PCA and OPLS-DA. **(A, C)**: Unsupervised Principal Component Analysis (PCA) used to examine the potential discriminatory potential of fecal analytes in the NA/RE **(A)** and the D2T/NA **(C)** (score plots represent individual patients and load plots represent individual analytes). **(B, D)**: Supervised Orthogonal Projection to Latent Structural Discriminant Analysis (OPLS-DA) to measure NA/RE **(B)** and D2T/NA **(D)** discriminatory ability based on fecal analytes (score plots representing individual patients) and to identify the specific analyte (s-plot) that drives the discrimination. OPLS-DA models discriminate between these groups of subjects confirm the validity of the model by permutation response testing (**Supplementary Figures 2A, B**). **(E, G)**: Unsupervised PCA to investigate the latent discrimination potential of plasma analytes in the NA/RE **(E)** and the D2T/NA **(G)** (scores plot representing individual patients, and loadings plot representing individual analytes). **(F, H)**: Supervised OPLS-DA to measure NA/RE **(F)** and D2T/NA **(H)** discrimination based on plasma analytes (scores plot representing individual patients) and to identify the specific analyte (s-plot) that drives the discrimination. OPLS-DA models discriminate between these groups of subjects confirm the validity of the model by permutation response testing (**Supplementary Figures 2C, D**). The OPLS-DA diagnosis then shows that the discriminant model is significant: cross-validation (CV) ANOVA p < 0.01; misclassification table Fisher’s p < 0.001.

Following the identification of independent regulation of fecal and plasma analytes in RA patients, we next determined whether fecal analytes profiling could be as a potential discriminatory biomarker among the four experimental groups through the application of multivariate analytical methods. Principal Component Analysis (PCA) and Orthogonal Partial Least Squares Discriminant Analysis (OPLS-DA) were performed on fecal and plasma analytes profile. PCA of fecal analytes effectively differentiated between NA and RE groups as well as between the D2T and NA groups (PC1 + 2 = 92.6% of the variance, PC’1 + 2 = 90.9% of the variance, **Figures 4A, C**). Plasma analytes were well-distinguished between the NA and RE groups and between the NA and D2T groups. (PC, **Figures 4E, G**). The fecal OPLS-DA model played a significant role in OPLS-DA diagnostics, enabling the identification of key discriminating factors such as D-lactate, zonulin, and FABP2 (**Figures 4B, D**). In contrast, the plasma-based OPLS-DA model identified plasma cytokines as the primary discriminating factors, highlighting distinct differences compared to fecal analytes (**Figures 4F, H**). The selection criteria included a VIP score > 1, an FC > 2 or < 0.5, and a P value < 0.05 (**Supplementary Figure 3**. **Supplementary Table 1**. **Supplementary Table 2**). The OPLS-DA models were evaluated based on their performance parameters, with R² > 0.8 and Q² > 0.8, demonstrating robust reliability and predictive validity (**Supplementary Figure 2** *).* Our findings delineate a pivotal divergence between fecal and plasma analytes and substantiate the earlier finding of separate co-regulation clusters in fecal and plasma data.

**Figure 5:**
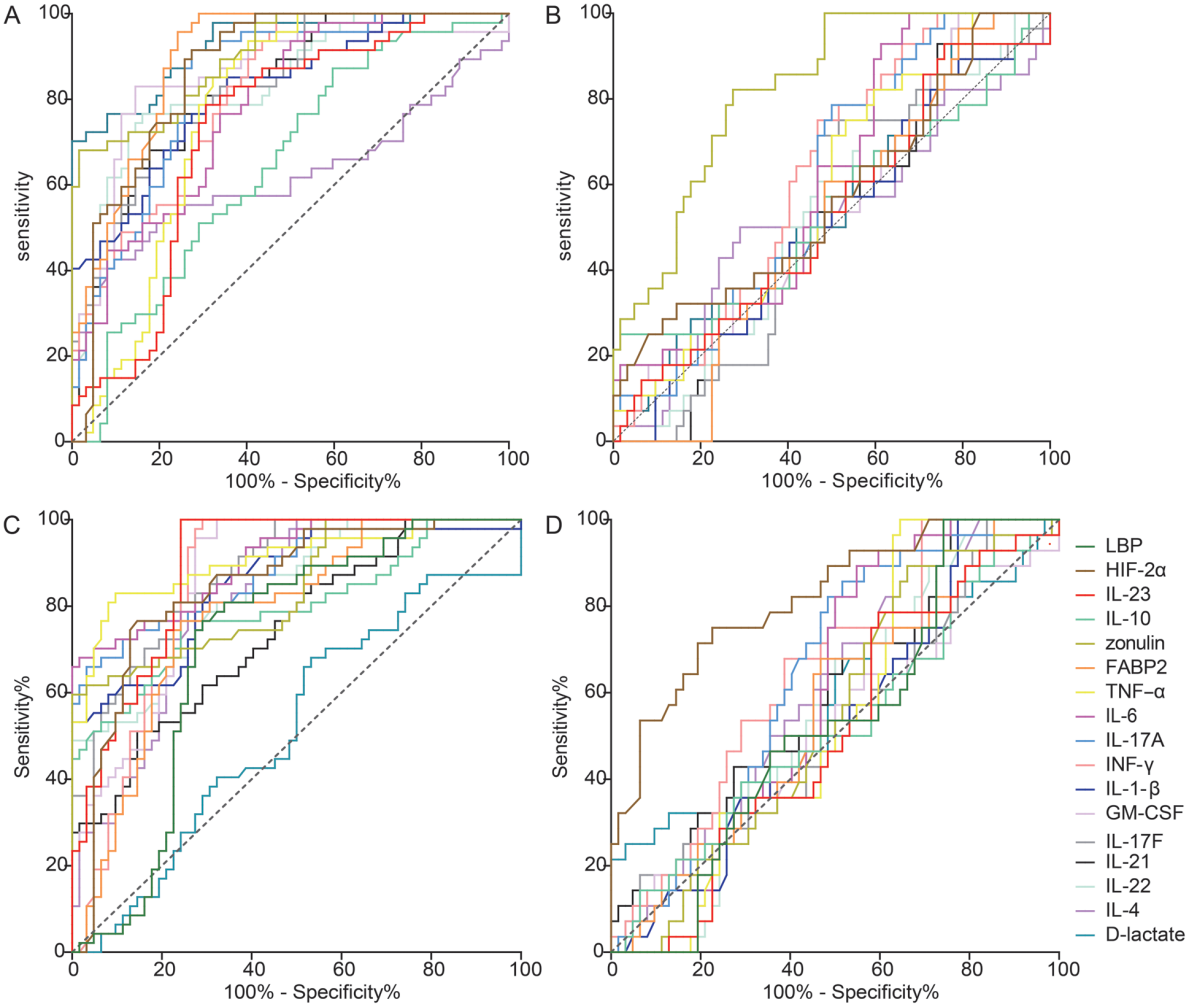
Receiver operating characteristic (ROC) curve analysis using fecal and plasma analytes to differentiate between patient groups. **(A)** ROC curves for the fecal naive and remission groups; **(B)** ROC curves for the fecal D2T and naive groups; **(C)** ROC curves for the plasma naive and remission groups; **(D)** ROC curves for the plasma D2T and naive groups.

To assess the differences in fecal analytes between the NA and RE groups as well as between the D2T and NA groups, ROC analyses were performed (**Figures 5A, B**). Fecal FABP2, zonulin and D-lactate were the most effective discriminators in the NA and RE groups (**Supplementary Table 3A**; Area Under the Curve(AUC) = 0.895, 0.901 and 0.925; p < 0.01). Subsequently, a linear combination of 16 significant fecal analytes (FABP2, D-lactate, zonulin, HIF-2α, IL-23, IL-21, IL-22, IL-17F, IL-1β, IL-4, IL-6, TNF-α, IL-10, IFN-γ, GM-CSF, and IL-17A) was utilized to create a discriminant score (DS) and its performance was assessed by AUC (**Supplementary Table 4A**; **Supplementary Figure 4A**). The DS model incorporating fecal FABP2, zonulin and D-lactate improved AUC to 0.9703 (0.9486-0.9920; q < 0.001), showing higher sensitivity (84%) and specificity (99%) compared to the use of these analytes alone. Thus further confirming that fecal FABP2, zonulin and D-lactate are key markers of intestinal barrier integrity and inflammation, which differentiate the NA group from the RE group. Similarly, fecal zonulin was the most effective discriminator between the D2T and NA groups (AUC=0.8237; p<0.01; sensitivity/specificity= 82.14%/72.58%. **Supplementary Table 3B** *)*, Moreover, only zonulin was statistically significant by the Wilcoxon rank-sum test.

Analysis of plasma analytes by the same method (**Figures 5C, D**) showed that, unlike the fecal markers, plasma FABP2, zonulin and D-lactate could not be used as valid discriminators. Plasma TNF-α was the most effective marker for differentiating the NA from the RE, with AUROC of 0.9061 (**Supplementary Table 3C**; p < 0.01; sensitivity/specificity = 82.98%/90.32%). The DS model constructed sequentially with 16 important plasma analytes used to differentiate the NA and RE groups showed that all 5 leading plasma parameters (TNF-α, IL-23, IL-6, IL-17A, and IL-17F) were necessary to achieve an optimal AUC of 0.9581 (**Supplementary Table 4B** *;* **Supplementary Figure 4B**; p < 0.001; sensitivity/specificity = 97.14%/92%), surpassing the performance of all other comparative models. Plasma HIF-2α was also the strongest discriminator between the D2T and NA *(* **Supplementary Table 3D**; AUC=0.8191; p < 0.01; sensitivity/specificity = 75%/77.42%), because the remaining plasma analytes were not significantly different between the two groups. Notably, the DS model based only on fecal analytes was superior to the optimal plasma cytokines model. The data indicated that fecal FABP2, D-lactate and zonulin might serve as potential discriminators between the NA and RE as well as between D2T and RE (**Supplementary Figure 5** *)*. Specially, fecal zonulin and plasma HIF-2α showed potential as discriminative biomarkers between the D2T and NA.

## Discussion

The functional integrity of the intestinal barrier is a pivotal factor in the pathophysiology of numerous intestinal and extraintestinal disorders. In this study, we reported the first profiling of fecal cytokines and biomarkers of intestinal barrier integrity in RA patients, offering critical insights into the interplay between gut mucosal immunity, barrier dysfunction, and disease processes. Our results provided evidence that fecal biomarkers, particularly involving the gut-joint axis, is significantly associated with RA and demonstrated a stronger correlation than comparable plasma biomarkers. Moreover, we observed that fecal cytokine levels and gut barrier integrity biomarkers were more sensitive in differentiating between the NA and RE groups with high specificity (86.67%) and sensitivity (98.29%), in contrast to the corresponding circulating plasma analytes. Likewise, comparative analysis revealed that fecal analytes achieved significantly higher sensitivity (82.14%) and specificity (72.58%) than plasma analytes in discriminating between the NA and D2T groups.

Emerging evidence underscores the critical role of fecal biomarkers in elucidating the gut-joint axis in RA. While serum biomarkers have been extensively studied, fecal biomarkers offer unique insights into gut dysbiosis and intestinal barrier dysfunction, which are increasingly implicated in RA pathogenesis (17). Dysbiosis characterized by altered microbial composition and metabolites in fecal samples has been linked to systemic inflammation, potentially driving autoimmune responses in RA (4). For instance, fecal microbial profiles may reflect shifts in pathogenic or protective bacterial communities, influencing disease progression and therapeutic outcomes (18). Notably, fecal biomarkers associated with gut permeability and microbial translocation could serve as early indicators of subclinical intestinal damage, preceding overt joint inflammation (12). This aligns with observations that interventions targeting gut integrity, such as probiotics or dietary modifications, modulate fecal microbial signatures and ameliorate arthritis severity (11). Furthermore, fecal biomarkers may predict treatment response, as therapies restoring gut homeostasis correlate with clinical improvement in RA patients (5). Collectively, fecal biomarkers hold promise as non-invasive tools for monitoring RA progression, stratifying patients, and guiding personalized therapeutic strategies, ultimately bridging the gap between gut pathophysiology and joint inflammation.

Prior investigations have elucidated an augmentation of intestinal permeability in early RA, and have delineated a correlation between fecal analytes and disease course as well as autoantibody production (3, 19, 20). The balance of gut mucosal homeostasis is increasingly linked to the activity of specific immune cell subsets (21). Currently, there is a pressing yet unmet need to identify biomarkers of gut inflammation and potential discriminators for predicting the various stage of RA. Fecal cytokine analysis has identified IL-2 and IFN-γ in norovirus-induced diarrhea and TNF-α in Crohn’s disease as potential biomarkers (22, 23). However, fecal cytokine profiling in RA remains to be determined. Our data indicated a widespread elevation in the levels of T-cell mediated type 17 effector cytokines in the feces of the NA and D2T groups, which may contribute to the exacerbation of gut mucosal inflammation in RA.

Additionally, fecal IFN-γ, IL-1β, IL-6, and TNF-α were higher in the NA and D2T groups compared to the RE and HC groups. These cytokines may promote inflammation through Th1 and Th17 pathways, commonly seen in chronic gut inflammation (14, 15, 24). These findings suggest the relevance of these pathways in RA-related gut damage. Cytokines play a dual role in mucosal immunity, exhibiting both pro-inflammatory and anti-inflammatory functions (24). Notably, cytokines such as IL-6, TNF-α, and IL-17A are primarily considered pro-inflammatory, they also promote epithelial cell proliferation, which is essential for wound healing and the replacement of cells lost due to homeostasis and potential pathological shedding (25–28). Furthermore, IL-22 is involved in the repair and protection of barrier surfaces, particularly in conjunction with IL-17A/F and IL-23, which collectively drives immune cell recruitment and activation during gut injury, as well as enhances barrier protection (29–32). However, IL-22 can also enhance the permeability of tight junctions in intestinal epithelial cells (IECs) and amplify the pro-inflammatory effects of TNF-α, contingent upon the surrounding microenvironment (32). Our study provides robust evidence supporting the critical involvement of fecal cytokines in the disease progression of RA, and these cytokines appear to originate predominantly from T cell-mediated adaptive immune processes. In contrast, the plasma cytokine profiles observed in RA seem to be principally regulated by innate immune processes. Therefore, targeting these cytokines in the systemic circulation may not represent the optimal therapeutic strategy. Further targeted studies are necessary to clarify the sources and mechanisms of action of these cytokines and to develop more effective and safer cytokine-targeted therapies for the treatment of patients with RA. Strikingly, fecal levels of IL-4 and IL-10 did not exhibit significant elevation in the NA or D2T groups relative to the RE or HC groups, a finding that contrasts with the observed increases in corresponding plasma analytes. This discrepancy may reflect an RA-specific immune response impairment, consistent with the immune dysfunction associated with RA, which may be impede the differentiation of anti-inflammatory T-regulatory cells; Neutralization of the cytokine IL-4 has been shown to restrict the cell differentiation of the intestinal epithelium and impair regenerative capacity of the intestinal mucosal barrier (33, 34). Overall, we observed a complex cytokine milieu that drives intestinal inflammation and perpetuates the disruption of the intestinal barrier in RA.

This study reinforces FCAL as a nonspecific inflammatory marker in RA, with elevated FCAL levels in NA patients correlating strongly with disease activity and systemic cytokines (13, 35). The lack of association between FCAL and fecal biomarkers suggests compartmentalized regulation, where FCAL may reflect gut-specific processes rather than systemic inflammation. This aligns with its established role in intestinal inflammation but underscores its limitations as a gut barrier marker in RA. Serum LBP, a putative indicator of intestinal barrier dysfunction, was also elevated in NA, implicating gut-derived microbial translocation in RA pathogenesis (36). However, LBP’s non-specificity—its levels may be confounded by extraintestinal factors such as tissue injury or drug effects—limits its utility as a standalone biomarker for intestinal permeability (37, 38). While our findings support a potential gut-joint axis in RA, the dual ambiguity of FCAL (systemic vs. gut inflammation) and LBP (barrier leakage vs. broader triggers) complicates definitive mechanistic interpretations. Key limitations include the cross-sectional design and absence of longitudinal or intestinal permeability comparator data. Future prospective studies incorporating metagenomic sequencing and standardized sampling protocols are needed.

Zonulin is a protein that plays a crucial role in regulating the permeability of the intestinal barrier, and helps in the normal physiological function of the gut (10, 39). However, when there is an aberration in the immune system or in the presence of certain diseases, the level of zonulin can change, leading to increased intestinal permeability, often referred to as “leaky gut” (10, 39, 40). Numerous studies have provided evidence for the association between zonulin and RA (3, 4, 10). Some research has shown that patients with pre-clinical signs of RA, such as elevated autoantibodies but no clinical symptoms yet, tend to have higher zonulin levels compared to healthy controls (10). In our study, fecal zonulin levels also differed among the groups, with the D2T group displaying significantly higher levels than both the NA and RE groups. Fecal levels of zonulin in all 4 groups were increased compared with their plasma counterparts. zonulin might serve as potential discriminators between NA and RE. Specially, fecal zonulin showed potential as discriminative biomarkers between D2T and NA. This finding implies that measurement of zonulin levels in feces has the potential to be used as a discriminatory tool to identify RA patients with high disease activity or refractory to treatment. In this context, the concept of zonulin as a biomarker is both novel and potentially transformative for the field of rheumatology.

The assessment of fecal FABP2 serves as a novel approach to indicate intestinal permeability and inflammation. This could signify the release of intestinal epithelial cells into the lumen, which may create temporary gaps or micro-erosions in the intestinal barrier, thereby increasing intestinal permeability and contributing to pathological bacterial translocation (17, 41). Elevated FABP2 usually indicates intestinal cellular damage. Even in the early phase, concentrations are elevated in response to increasing damage and basal FABP2 levels may reflect the physiologic turnover rate of intestinal epithelial cells (42, 43). Fecal FABP2 was significantly higher in the NA and D2T groups, suggesting that intestinal barrier damage is associated with RA disease progression. Our study demonstrated that although fecal levels were significantly lower than plasma levels, fecal FABP2 was more sensitive than plasma FABP2 in identifying the NA and RE. Our study was a cross-sectional single time-point collection, and more detailed studies should be conducted longitudinally in patients to clarify whether FABP2 is a reliable biomarker.

D-lactate is a metabolite produced by intestinal flora and is used as a biomarker to assess intestinal permeability. Elevated serum D-lactate concentrations can be used to confirm intestinal barrier damage and translocation of microorganisms and their metabolites, which has been demonstrated in inflammatory bowel disease, intestinal ischemia and advanced cirrhosis (9, 44, 45). However, in our study we demonstrated that plasma D-lactate was not significantly differentiated among the groups. Notably, we found that fecal D-lactate levels were lower in the NA and D2T than in the RE. We hypothesize that this may be related to the following mechanisms: first, increased transport from the intestinal lumen to the systemic circulation through the compromised intestinal barrier; second, lower fecal levels of D-lactate metabolites in the naive group due to an enrichment of intestinal microbial species metabolizing D-lactate as a result of dysbiosis; and a decrease in the number of intestinal bacterial species (e.g., Lactobacillus delbrueckii and Leuconostoc spp.) producing D-lactate. The pathogenesis and metabolism of D-lactate in RA need to be studied more closely.

HIF-2α expression peaks in intestinal epithelial cells during arthritis onset in both murine models and RA patients (46). Notably, conditional deletion of HIF-2α in intestinal epithelial cells attenuates arthritis severity, suggesting that gut epithelial HIF-2α activation is not merely a bystander effect but a driver of disease progression (47, 48). Mechanistically, HIF-2α transcriptionally upregulates claudin-15, a pore-forming tight junction protein linked to increased intestinal permeability (47). This disruption allows microbial translocation, triggering systemic immune activation. In intestinal epithelial cell-specific HIF-2α conditional knock-out mice, reduced Th17 infiltration in joints correlates with diminished arthritis severity (47, 49). Our analysis of plasma from RA subjects has shown a positive correlation between HIF-2α levels and disease severity indices. Fecal analysis also revealed higher levels of HIF-2α in RA compared to healthy controls. Intriguingly, fecal zonulin and plasma HIF-2α showed potential as discriminative biomarkers between D2T and NA through multivariate analysis. These findings suggest that HIF-2α in plasma and fecal analytes can serve as biomarkers of immune cell activation and crosstalk, which are crucial processes in the development of RA.

Our study systematically quantified cytokines and intestinal barrier markers (e.g., FABP2, D-lactate, etc.) in feces, providing a non-invasive assessment tool for RA intestinal inflammation and compensating for the limitations of difficult-to-access tissue biopsies. In addition, this study simultaneously detected 18 cytokines and barrier markers in feces and plasma, revealing differences between local and systemic inflammatory responses in the gut and combining multivariate statistics (PCA, OPLS-DA) and ROC curves to derive valuable discriminatory factors. However, our study has limitations. First, its observational design prevents establishing causal links between gut microbiota changes and RA progression. Without experimental control, we can’t determine cause- and -effect; Second, the cohort size, suitable for exploratory analysis, may limit result generalizability as it might not fully represent the entire RA-affected population; Third, although we adjusted for common confounders, unmeasured variables like diet and genetic background could still bias the results and impact the study’s validity; Another limitation is the use of GCs, especially in patients naive to DMARDs and constituting the csDMARD-treated groups. GCs are almost universally used concomitantly with other DMARDs, making it difficult to assess the effects of GCs on this study validity alone.

Despite long-term treatment, persistent disease activity and inflammation in D2T RA may stem from a complex interplay of high baseline autoimmunity (RF/ACPA), multi-pathway immune activation (TNF-α/IL-6/JAK-STAT), comorbidities, and socioeconomic barriers limiting optimal therapy access (50–54). Collectively, analyzing cytokines and gut barrier integrity markers in feces offers a new way to assess the intestinal cytokine micro-environment in RA, evaluating gut inflammation and barrier function simultaneously. Our study has demonstrated the significance of biomarkers of intestinal barrier integrity and inflammation in relation to the prognosis of RA across different stages, and the profiles of fecal cytokines and gut integrity markers, likely T-cell-driven, differ greatly from systemic inflammation markers in RA detected by plasma assays. These findings not only contribute to the existing knowledge of the pathophysiology of RA but also hold promise for the development of novel diagnostic and prognostic tools, as well as more targeted therapeutic strategies for RA patients.

This study assessed intestinal mucosal inflammation and barrier dysfunction by analyzing cytokines and intestinal permeability markers. Further cellular-level research is needed to elucidate the poorly understood mechanisms underlying RA. Clarifying cytokine biology may advance novel therapeutic development, while the effects of gut-targeted therapies on fecal cytokine production warrant additional investigation.

## Data Availability

The datasets presented in this study can be found in online repositories. The names of the repository/repositories and accession number(s) can be found in the article/**Supplementary Material**.
