# Supplementary figures and images for: Profiling of fecal analytes as a potential biomarker in rheumatoid arthritis
Source: Front Immunol. 2025 May 19;16:1577590. doi: 10.3389/fimmu.2025.1577590 (PMC12127413; doi:10.3389/fimmu.2025.1577590)

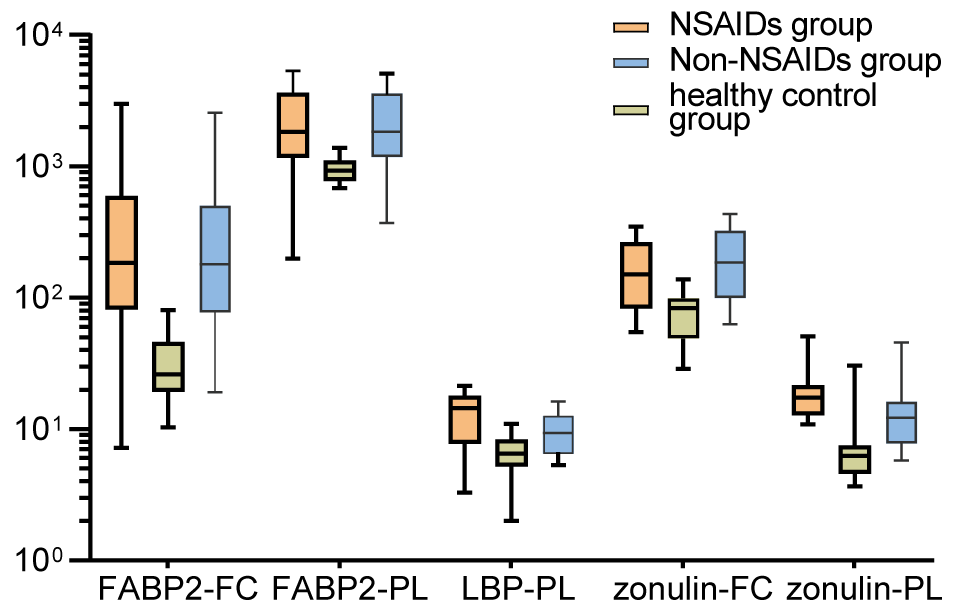

Supplement: Supplementary file 1 [file Image1.tif]

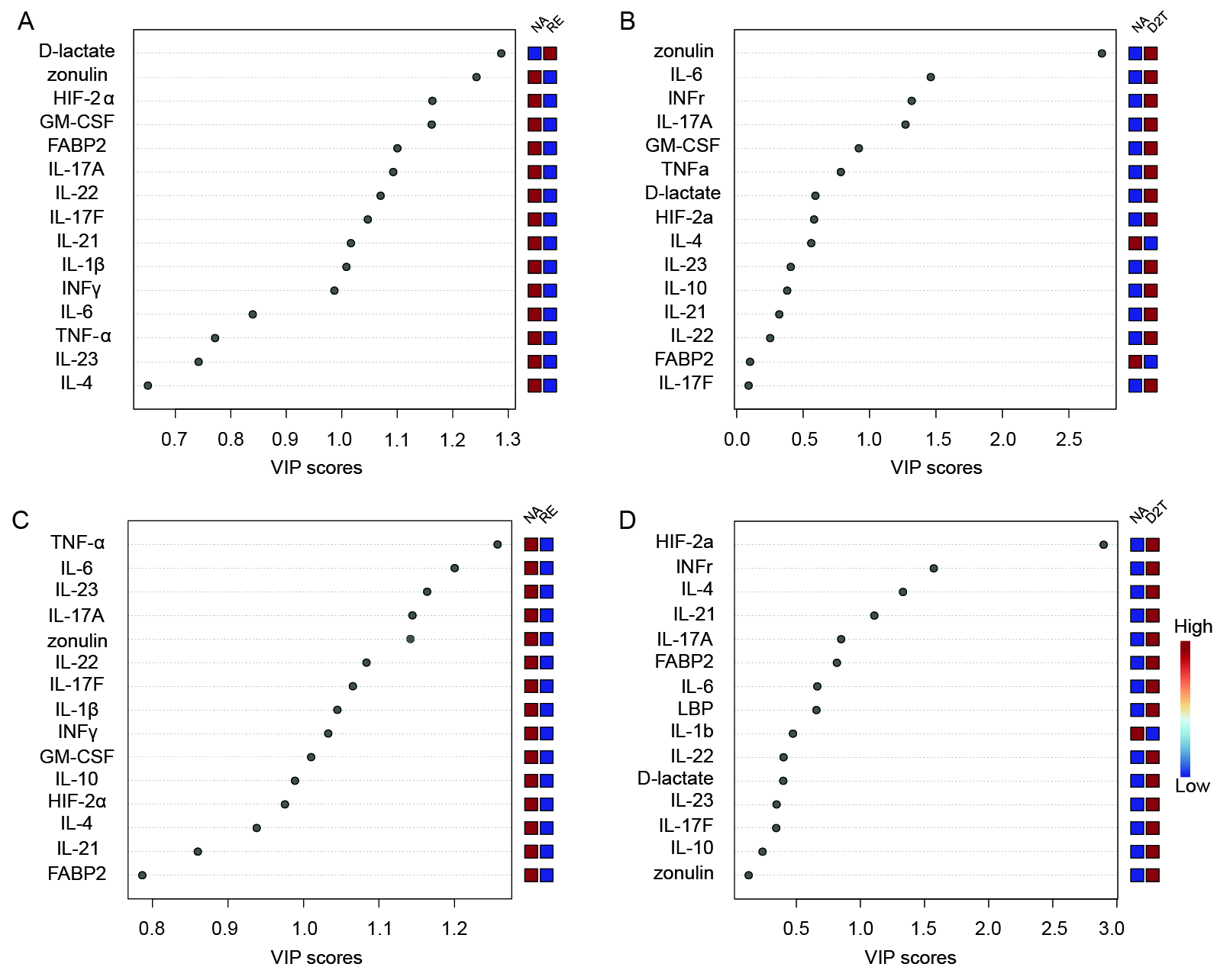

Supplement: Supplementary file 3 [file Image3.tif]

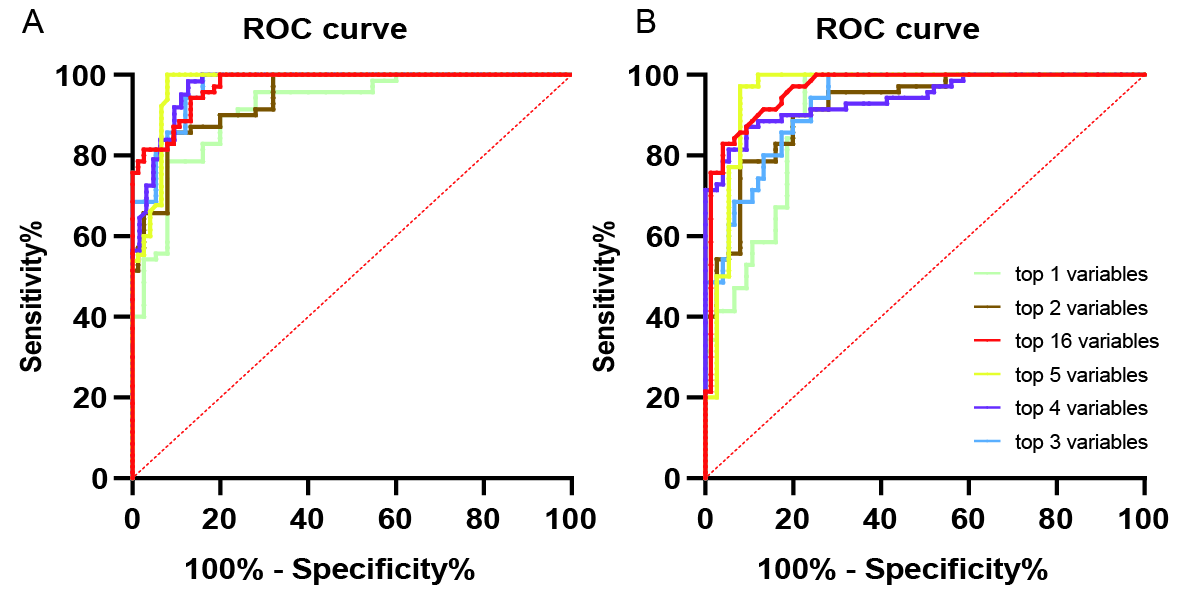

Supplement: Supplementary file 4 [file Image4.tif]

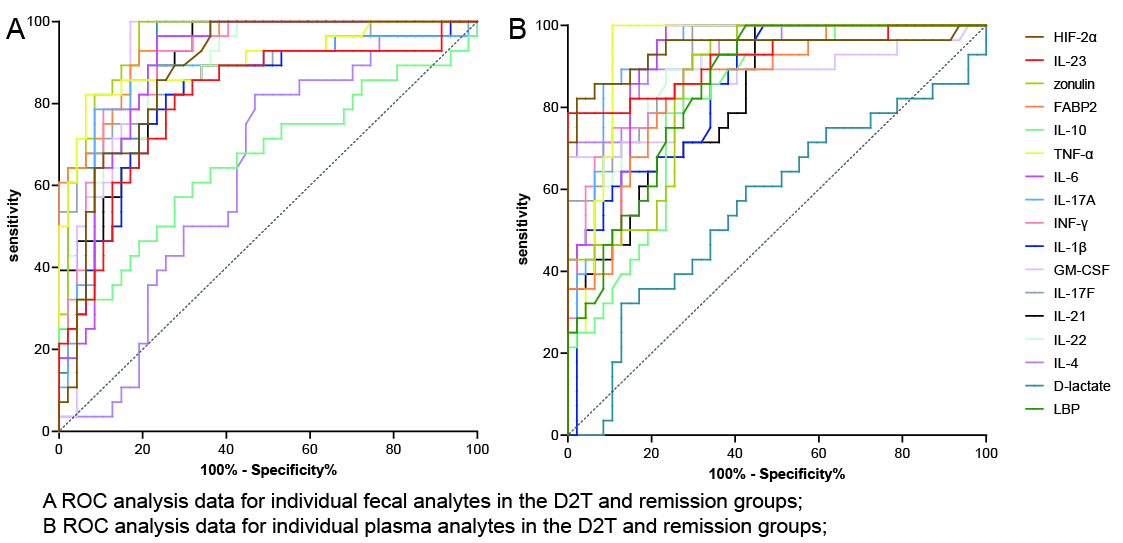

Supplement: Supplementary file 5 [file Image5.tif]
