# Supplementary material for: Profiling of fecal analytes as a potential biomarker in rheumatoid arthritis
Source: Front Immunol. 2025 May 19;16:1577590. doi: 10.3389/fimmu.2025.1577590 (PMC12127413; doi:10.3389/fimmu.2025.1577590)
Supplement: Supplementary file 6 [file Table1.docx]

**Supplementary Table S1:** **Rank-sum test between NA and RE groups, D2T and NA groups in plasma and fecal analytes**

1. Rank-sum test for individual fecal analytes in the naive and remission groups

|  | **V** | **p-value** | **LOG10(p)** | **FDR** |
| --- | --- | --- | --- | --- |
| **Dlactate** | 216 | 3.1971e-14 | 13.495 | 5.1154e-13 |
| **zonulin** | 2627.5 | 8.1424e-13 | 12.089 | 6.5139e-12 |
| **FABP2** | 2609.5 | 1.8068e-12 | 11.743 | 9.6364e-12 |
| **HIF2a** | 2532 | 4.8836e-11 | 10.311 | 1.9534e-10 |
| **GMCSF** | 2487 | 2.9942e-10 | 9.5237 | 9.5816e-10 |
| **IL22** | 2452.5 | 1.1434e-09 | 8.9418 | 3.0491e-09 |
| **IL17A** | 2441 | 1.7701e-09 | 8.752 | 4.046e-09 |
| **IL17F** | 2409 | 5.8212e-09 | 8.235 | 1.1549e-08 |
| **IL21** | 2406 | 6.4961e-09 | 8.1873 | 1.1549e-08 |
| **IL1b** | 2384 | 1.4374e-08 | 7.8424 | 2.2998e-08 |
| **INFr** | 2376 | 1.9107e-08 | 7.7188 | 2.7792e-08 |
| **IL6** | 2285 | 4.1247e-07 | 6.3846 | 5.4996e-07 |
| **TNFa** | 2248.5 | 1.2997e-06 | 5.8861 | 1.5997e-06 |
| **IL23** | 2119 | 5.1783e-05 | 4.2858 | 5.9181e-05 |
| **IL10** | 1889.5 | 0.0082119 | 2.0856 | 0.0087593 |
| **IL4** | 1809.5 | 0.03126 | 1.505 | 0.03126 |

1. Rank-sum test for individual plasma analytes in the naive and remission groups

|  | **V** | **p-value** | **LOG10(p)** | **FDR** |
| --- | --- | --- | --- | --- |
| **TNF-a** | 2640.5 | 4.5442e-13 | 12.343 | 5.6597e-12 |
| **IL-6** | 2632 | 6.6585e-13 | 12.177 | 5.6597e-12 |
| **IL-23** | 2590 | 4.2301e-12 | 11.374 | 2.397e-11 |
| **IL-17A** | 2564.5 | 1.2587e-11 | 10.9 | 5.3493e-11 |
| **IL-17F** | 2554 | 1.9596e-11 | 10.708 | 6.6625e-11 |
| **GM-CSF** | 2505 | 1.4626e-10 | 9.8349 | 4.1441e-10 |
| **IL-1b** | 2481 | 3.7837e-10 | 9.4221 | 9.189e-10 |
| **INFr** | 2473 | 5.184e-10 | 9.2853 | 1.1016e-09 |
| **HIF-2a** | 2464.5 | 7.2023e-10 | 9.1425 | 1.3604e-09 |
| **zonulin** | 2439.5 | 1.8726e-09 | 8.7275 | 3.1835e-09 |
| **IL-22** | 2436 | 2.1369e-09 | 8.6702 | 3.3025e-09 |
| **IL-4** | 2371.5 | 2.2398e-08 | 7.6498 | 3.173e-08 |
| **IL-10** | 2319 | 1.3548e-07 | 6.8681 | 1.7716e-07 |
| **FABP2** | 2263.5 | 8.157e-07 | 6.0885 | 9.9049e-07 |
| **IL-21** | 2159.5 | 1.7448e-05 | 4.7583 | 1.9774e-05 |
| **LBP** | 2070.5 | 0.00017635 | 3.7536 | 0.00018737 |

1. Rank-sum test for individual fecal analytes in the naive and D2T groups

|  | **V** | **p.value** | **LOG10(p)** | **FDR** |
| --- | --- | --- | --- | --- |
| **zonulin** | 306 | 9.8915e-07 | 6.0047 | 1.5826e-05 |

1. Rank-sum test for individual plasma analytes in the naive and D2T groups

|  | **V** | **p.value** | **LOG10(p)** | **FDR** |
| --- | --- | --- | --- | --- |
| **HIF-2a** | 314 | 1.4051e-06 | 5.8523 | 2.3887e-05 |

**Supplementary Table S2:** **Fold Change Analysis between NA and RE groups, D2T and NA groups in plasma and fecal analytes**

1. Fold Change Analysis for individual fecal analytes in the naive and remission groups

|  | **Fold Change** | **log2(FC)** |
| --- | --- | --- |
| **FABP2** | 55.131 | 5.7848 |
| **HIF2a** | 7.4362 | 2.8946 |
| **IL17F** | 5.6604 | 2.5009 |
| **IL21** | 4.5095 | 2.173 |
| **IL22** | 4.1858 | 2.0655 |
| **IL17A** | 4.184 | 2.0649 |
| **TNFa** | 4.1802 | 2.0636 |
| **IL1b** | 3.9826 | 1.9937 |
| **INFr** | 3.2096 | 1.6824 |
| **GMCSF** | 3.0799 | 1.6229 |
| **IL23** | 2.7388 | 1.4535 |
| **zonulin** | 2.6638 | 1.4135 |
| **IL6** | 2.417 | 1.2732 |

1. Fold Change Analysis for individual plasmal analytes in the naive and remission groups

|  | **Fold Change** | **log2(FC)** |
| --- | --- | --- |
| **IL-6** | 11.828 | 3.5642 |
| **IL-17A** | 11.715 | 3.5503 |
| **INFr** | 10.417 | 3.3809 |
| **IL-22** | 7.4907 | 2.9051 |
| **TNF-a** | 6.8084 | 2.7673 |
| **IL-17F** | 5.2455 | 2.3911 |
| **HIF-2a** | 3.8123 | 1.9307 |
| **IL-10** | 3.2867 | 1.7166 |
| **IL-23** | 3.1249 | 1.6438 |
| **IL-21** | 2.6202 | 1.3897 |
| **IL-4** | 2.5969 | 1.3768 |
| **GM-CSF** | 2.5128 | 1.3293 |
| **zonulin** | 2.3664 | 1.2427 |
| **LBP** | 2.2478 | 1.1685 |
| **FABP2** | 2.1984 | 1.1364 |

1. Fold Change Analysis for individual fecal analytes in the naive and D2T groups

|  | **Fold Change** | **log2(FC)** |
| --- | --- | --- |
| **FABP2** | 3.1681 | 1.6636 |

1. Fold Change Analysis for individual plasma analytes in the naive and D2T groups

|  | **Fold Change** | **log2(FC)** |
| --- | --- | --- |
| **HIF-2a** | 0.27047 | -1.8865 |
| **IL-1b** | 2.075 | 1.0531 |

**Supplementary Table S3: Receptor Operating Characterization (ROC) Analysis Data for Individual fecal and plasma Analytes.**

1. ROC analysis data for individual fecal analytes in the naive and remission groups;

| Test Result Variable(s) | Area Under the Curve | p-value | Cut-off | Sensitivity | Specificity |
| --- | --- | --- | --- | --- | --- |
| zonulin | 0.9017(0.8471-0.9563) | <0.01 | 156.0 | 68.09 | 98.39 |
| FABP2 | 0.8955(0.8377-0.9533) | <0.01 | 58.71 | 95.74 | 75.81 |
| D-lactate | 0.9259(0.8769-0.9748) | <0.01 | 509.5 | 70.21 | 100 |
| IL-17A | 0.8377(0.7632-0.9121) | <0.01 | 131.9 | 91.49 | 72.58 |
| IL-21 | 0.8257(0.7499-0.9014) | <0.01 | 93.16 | 76.6 | 74.19 |
| IL-22 | 0.8416(0.7671-0.9161) | <0.01 | 23.38 | 76.6 | 83.87 |
| IL-17F | 0.8267（0.7519-0.9015） | <0.01 | 7660 | 82.98 | 66.13 |
| IL-1β | 0.8181(0.7395-0.8967) | <0.01 | 36.14 | 85.11 | 64.52 |
| INFγ | 0.8154(0.7386-0.8921) | <0.01 | 539.0 | 100 | 53.23 |
| TNF-α | 0.7716(0.6817-0.8615) | <0.01 | 76.14 | 93.62 | 59.68 |
| IL-6 | 0.7841(0.7008-0.8675) | <0.01 | 55.04 | 87.23 | 56.45 |
| HIF-2α | 0.8689(0.8012-0.9366） | <0.01 | 6.89 | 91.49 | 72.58 |
| IL-23 | 0.7272(0.6307-0.8237) | <0.05 | 4447 | 78.72 | 69.35 |
| GM-CSF | 0.8535(0.7758-0.9312) | <0.01 | 42.27 | 82.98 | 85.48 |
| IL-4 | 0.6210(0.5040-0.7366) | <0.05 | 34.87 | 42.55 | 90.32 |
| IL-10 | 0.6484(0.5455-0.7513) | <0.05 | 54.88 | 87.23 | 40.32 |

1. ROC analysis data for individual fecal analytes in the naive and D2T groups;

| Test Result Variable(s) | Area Under the Curve | p-value | Cut-off | Sensitivity | Specificity |
| --- | --- | --- | --- | --- | --- |
| zonulin | 0.8237(0.7391 to 0.9084) | <0.01 | 416.8 | 82.14 | 72.58 |
| IL-4 | 0.5239（0.3881-0.6597） | >0.05 | - | - | - |
| INFγ | 0.6215（0.5071-0.7360） | >0.05 | - | - | - |
| HIF-2α | 0.5726（0.4419-0.7032） | >0.05 | - | - | - |
| IL-1β | 0.5092（0.3823-0.6361） | >0.05 | - | - | - |
| TNF-α | 0.5804（0.4595-0.7012） | >0.05 | - | - | - |
| IL-6 | 0.6141（0.4952-0.7329） | >0.05 | - | - | - |
| D-lactate | 0.5588（0.4335-0.6840） | >0.05 | - | - | - |
| IL-21 | 0.5161(0.3961-0.6361) | >0.05 | - | - | - |
| IL-10 | 0.5432(0.4059-0.6805) | >0.05 | - | - | - |
| IL-17A | 0.6074(0.4903-0.7246) | >0.05 | - | - | - |
| IL-23 | 0.5323(0.4040-0.6605) | >0.05 | - | - | - |
| IL-17F | 0.5161(0.3936-0.6386) | >0.05 | - | - | - |
| GM-CSF | 0.5487(0.4235-0.6738) | >0.05 | - | - | - |
| FABP2 | 0.5141(0.3941-0.6341) | >0.05 | - | - | - |
| IL-22 | 0.5317(0.4089-0.6545) | >0.05 | - | - | - |

1. ROC analysis data for individual plasma analytes in the naive and remission groups;

| Test Result Variable(s) | Area Under the Curve | p-value | Cut-off | Sensitivity | Specificity |
| --- | --- | --- | --- | --- | --- |
| TNF-α | 0.9061(0.8469-0.9654) | <0.01 | 24.80 | 82.98 | 90.32 |
| IL-6 | 0.9032(0.8483-0.9582) | <0.01 | 17.40 | 68.09 | 98.39 |
| IL-17A | 0.8801(0.8159-0.9443) | <0.01 | 17.54 | 65.96 | 96.77 |
| IL-23 | 0.8888(0.8287-0.9489) | <0.01 | 11035 | 100 | 75.81 |
| IL-17F | 0.8765(0.8151-0.9378) | <0.01 | 815.5 | 80.85 | 75.81 |
| HIF-2α | 0.8457(0.7702-0.9213) | <0.01 | 0.82 | 76.6 | 85.48 |
| IL-10 | 0.7958(0.7078-0.8838) | <0.01 | 4.540 | 76.6 | 75.81 |
| INFγ | 0.8487(0.7736-0.9237) | <0.01 | 100.8 | 100 | 70.97 |
| IL-4 | 0.8138(0.7359-0.8917) | <0.01 | 15.20 | 76.6 | 74.19 |
| GM-CSF | 0.8596(0.7914-0.9279) | <0.01 | 43.62 | 95.74 | 72.58 |
| LBP | 0.7105(0.6104-0.8107) | <0.01 | 12.13 | 76.6 | 70.97 |
| IL-21 | 0.7411(0.6492-0.8330) | <0.01 | 4.625 | 51.06 | 85.48 |
| IL-1β | 0.8514(0.7790-0.9238) | <0.01 | 0.200 | 53.19 | 100 |
| zonulin | 0.8372(0.7611-0.9132) | <0.01 | 10.74 | 59.57 | 100 |
| IL-22 | 0.8360(0.7630-0.9089) | <0.01 | 27.55 | 76.6 | 72.58 |
| FABP2 | 0.7768(0.6891-0.8644) | <0.01 | 1514 | 76.6 | 75.81 |
| D-lactate | 0.5180(0.4076-0.6284) | >0.05 | - | - | - |

1. ROC analysis data for individual plasma analytes in the naive and D2T groups;

| Test Result Variable(s) | Area Under the Curve | p-value | Cut-off | Sensitivity | Specificity |
| --- | --- | --- | --- | --- | --- |
| HIF-2α | 0.8191(0.7257-0.9126) | <0.01 | 5.10 | 75 | 77.42 |
| IL-6 | 0.5910(0.4768-0.7052) | >0.05 | - | - | - |
| FABP2 | 0.5573(0.4341-0.6806) | >0.05 | - | - | - |
| D-lactate | 0.5709(0.4345-0.7072) | >0.05 | - | - | - |
| IL-21 | 0.6002(0.4778-0.7226) | >0.05 | - | - | - |
| IL-1β | 0.5294(0.4064-0.6523) | >0.05 | - | - | - |
| GM-CSF | 0.5262(0.3945-0.6579) | >0.05 | - | - | - |
| IL-10 | 0.5193(0.3883-0.6503) | >0.05 | - | - | - |
| LBP | 0.5262(0.4050-0.6474) | >0.05 | - | - | - |
| TNF-α | 0.5446(0.4272-0.6621) | >0.05 | - | - | - |
| IL-17F | 0.5308(0.4036-0.6580) | >0.05 | - | - | - |
| IL-23 | 0.5040(0.3814-0.6267) | >0.05 | - | - | - |
| zonulin | 0.5415(0.4210-0.6620) | >0.05 | - | - | - |
| IL-17A | 0.6431(0.5297-0.7566) | 0.0303 | 45.69 | 85.71 | 48.39 |
| IL-4 | 0.6077(0.4892-0.7262) | >0.05 | - | - | - |
| IL-22 | 0.5504(0.4324-0.6684) | >0.05 | - | - | - |
| INFγ | 0.6457(0.5293-0.7622) | 0.0275 | 103.00 | 100 | 30.65 |

**Supplementary Table S4: Receiver Operating Characteristic (ROC) analysis data for Discriminant Score (DS) models** built using linear combinations of the most significant fecal and plasma analytes as listed in Table S4.

(A)

| Test Result Variable(s) | Area Under the Curve | p-value | Cut-off DS | Sensitivity | Specificity |
| --- | --- | --- | --- | --- | --- |
| DS (top 16 variables) | 0.9739(0.9548 - 0.9930) | <0.001 | -0.55 | 0.8667 | 0.9829 |
| DS (top 3 variables) | 0.9703(0.9486 - 0.9920) | <0.001 | -0.81 | 0.84 | 0.99 |
| DS (top 5 variables) | 0.9729(0.9490-0.9969) | <0.001 | -0.79 | 1 | 0.92 |
| DS (top 4 variables) | 0.9722(0.9487 - 0.9957) | <0.001 | -0.8 | 98.39 | 87.3 |
| DS (top 2 variables) | 0.9415 (0.9077 - 0.9753) | <0.001 | -0.79 | 85.71 | 90.67 |
| DS (top 1 variables) | 0.9164(0.8725-0.9603) | <0.001 | -0.83 | 78.57 | 92 |

(B)

| Test Result Variable(s) | Area Under the Curve | p-value | Cut-off DS | Sensitivity | Specificity |
| --- | --- | --- | --- | --- | --- |
| DS (top 16 variables) | 0.9647(0.9374 - 0.9919) | <0.001 | -0.69 | 82.86 | 96 |
| DS (top 5 variables) | 0.9581(0.9226 - 0.9936) | <0.001 | -0.80 | 97.14 | 92 |
| DS (top 4 variables) | 0.9413(0.9046 - 0.9780) | <0.001 | -0.74 | 87.14 | 90.67 |
| DS (top 3 variables) | 0.9299(0.8919 - 0.9679) | <0.001 | -0.84 | 100 | 72 |
| DS (top 2 variables) | 0.9187(0.8759 - 0.9615) | <0.001 | -0.81 | 78.57 | 92 |
| DS (top 1 variables) | 0.9027(0.8531-0.9523) | <0.001 | -0.88 | 100 | 77.33 |

(A) Discriminant score (DS) ROC analysis data for fecal naive and remission groups;

(B) Discriminant score (DS) ROC analysis data for plasma naive and remission groups. ROC curves corresponding to these data are in Fig. S4.
